# Incidence and Prognosis of Acute Kidney Diseases and Disorders Using an Integrated Approach to Laboratory Measurements in a Universal Health Care System

**DOI:** 10.1001/jamanetworkopen.2019.1795

**Published:** 2019-04-05

**Authors:** Matthew T. James, Andrew S. Levey, Marcello Tonelli, Zhi Tan, Rebecca Barry, Neesh Pannu, Pietro Ravani, Scott W. Klarenbach, Braden J. Manns, Brenda R. Hemmelgarn

**Affiliations:** 1 Department of Medicine, Cumming School of Medicine, University of Calgary, Alberta, Canada; 2 Libin Cardiovascular Institute of Alberta, Cumming School of Medicine, University of Calgary, Alberta, Canada; 3 Department of Community Health Sciences, Cumming School of Medicine, University of Calgary, Alberta, Canada; 4 O’Brien Institute for Public Health, Cumming School of Medicine, University of Calgary, Alberta, Canada; 5 Tufts Medical Center, Boston, Massachusetts; 6 Department of Medicine, University of Alberta, Alberta, Canada

## Abstract

**Importance:**

Abnormal measurements of kidney function or structure may be identified that do not meet criteria for acute kidney injury (AKI) or chronic kidney disease (CKD) but nonetheless may require medical attention. The Kidney Disease: Improving Global Outcomes Clinical Practice Guideline for AKI proposed criteria for the definition of acute kidney diseases and disorders (AKD), which include AKI; however, the incidence and prognosis of AKD without AKI remain unknown.

**Objective:**

To characterize the incidence and outcomes of AKD without AKI, with or without CKD.

**Design, Setting, and Participants:**

Retrospective cohort study including all adult residents in a universal health care system in Alberta, Canada, without end-stage kidney disease (ESKD) and with at least 1 serum creatinine measurement between January 1 and December 31, 2008, in a community or hospital setting. Data analysis took place in 2018.

**Main Outcomes and Measures:**

The Kidney Disease: Improving Global Outcomes guideline definitions for CKD, AKI, and AKD based on serum creatinine, estimated glomerular filtration rate, and albuminuria criteria were applied to estimate the proportion of patients with CKD, AKI, and AKD without AKI, and combinations of the conditions. Patients were followed up for up to 8 years (study end date, June 31, 2016) to characterize their risks of mortality, development of new CKD, progression of preexisting CKD, and ESKD.

**Results:**

Among 1 109 099 Alberta residents included in the cohort, the mean (SD) age was 52.3 (17.6) years, and 43.0% were male. Findings showed that AKD without AKI was common (3.8 individuals without preexisting CKD and 0.6 with preexisting CKD per 100 population tested). In Cox proportional hazards and competing risks models over a median (interquartile range) of 6.0 (5.7-6.3) years of follow-up, AKD without AKI (compared with no kidney disease) was associated with higher risks of developing new CKD (37.4% vs 7.4%%; adjusted sub–hazard ratio [sHR], 3.17; 95% CI, 3.10-3.23), progression of preexisting CKD (49.5% vs 34.6%; adjusted sHR, 1.38; 95% CI, 1.33-1.44), ESKD (0.6% vs 0.1%; adjusted sHR, 8.56; 95% CI, 7.32-10.01), and death (25.8% vs 7.3%; adjusted hazard ratio, 1.42; 95% CI, 1.39-1.45).

**Conclusions and Relevance:**

Criteria for AKD identified many patients who did not meet the criteria for CKD or AKI but had overall modestly increased risks of incident and progressive CKD, ESKD, and death. The clinical importance of AKD remains to be determined.

## Key Points

**Question:**

Do the acute kidney diseases and disorders (AKD) criteria from the Kidney Disease: Improving Global Outcomes guidelines identify patients who do not meet existing criteria for chronic kidney disease (CKD) or acute kidney injury (AKI), and what is the prognosis of these patients?

**Findings:**

In this cohort study of 1.1 million Canadian residents, AKD criteria identified many patients who did not meet existing criteria for CKD or AKI, and patients with AKD without AKI had overall modestly increased risks of incident and progressive CKD, end-stage kidney disease, and death.

**Meaning:**

The incorporation of AKD into clinical and research initiatives for kidney disease would increase recognition of patients at risk of adverse outcomes who are not identified by current AKI and CKD criteria; however, the clinical importance of AKD remains to be determined.

## Teaser

This cohort study investigates whether the Kidney Disease: Improving Global Outcomes guidelines can identify patients with acute kidney diseases and disorders who do not meet existing criteria for chronic kidney disease or acute kidney injury and whether these patients experience adverse outcomes.

## Introduction

Kidney function is assessed routinely in clinical practice to detect acute kidney injury (AKI) and chronic kidney disease (CKD); AKI is defined as decline in kidney function over 1 week or less, while CKD is defined as alteration of kidney function and structure for more than 3 months.^1,2,3,4^ However, alterations in kidney function or structure are frequently detected in acute and chronic illness that do not meet the criteria for CKD or AKI but nonetheless may require medical attention. Recognizing this, the 2012 Kidney Disease: Improving Global Outcomes (KDIGO) Clinical Practice Guideline for AKI proposed an operational definition for acute kidney diseases and disorders (AKD) as alterations in kidney function or structure for less than 3 months, which includes AKI (eTable 1 in the Supplement).^3^

Identification of AKD, in addition to AKI and CKD, could enable creation of standardized diagnostic algorithms for prompt recognition and appropriate clinical evaluation of these disorders and facilitate comprehensive research, surveillance, and public health initiatives for kidney diseases. However, further research to characterize the incidence and outcomes of AKD has been recommended before adoption, as very few studies to date have evaluated the incremental value of AKD in epidemiological research on kidney disease.^5^

The purpose of this study was to characterize the frequency and prognosis of AKD without AKI across a geographic region with universal access to health care and clinical laboratory testing that included serum creatinine (sCr) and albuminuria measurements from all adult residents of the province of Alberta, Canada. We sought to compare the frequency of identification of AKD without AKI with that of CKD, AKI, and combinations of these conditions, as well as to compare the prognosis of these groups for the risks of development or progression of CKD or end-stage kidney disease (ESKD) and risk of death.

## Methods

We followed the Strengthening the Reporting of Observational Studies in Epidemiology (STROBE) reporting guideline.^6^ The Conjoint Health Research Ethics Board of the University of Calgary approved the study and granted waiver of patient consent.

### Cohort Formation

Outpatient and inpatient laboratory and administrative data with comprehensive coverage of all residents of the province of Alberta were linked as previous described.^7^ The study population included all residents of Alberta aged 18 years and older with at least 1 sCr measurement obtained between January 1 and December 31, 2008. The first sCr measurement in 2008 was identified for each individual, and the index date was defined as the date of this measurement. Patients with estimated glomerular filtration rate (eGFR) less than 15 mL/min/1.73 m^2^ or who were receiving treatment for ESKD prior to the index date, identified by physician claims or records for dialysis or a prior kidney transplant in the Northern or Southern or Alberta Renal programs,^8,9^ were excluded from the cohort.

### Ascertainment of CKD, AKI, and AKD Without AKI

All inpatient and outpatient sCr measurements from January 1, 2004, to June 30, 2016, were identified from laboratories using isotope-dilution mass spectroscopy standardized creatinine assays, and eGFR was calculated using the Chronic Kidney Disease Epidemiology Collaboration equation.^10^ Information on black race was not available; however, misclassification was expected to be minimal, as less than 1% of the Alberta population is of African descent. Albuminuria measurements over the same period were categorized based on results from random urine albumin to creatinine ratio (ACR) or urine dipstick testing as unmeasured, none (ACR <3 mg/mmol or dipstick negative), moderate (ACR 3-30 mg/mmol or dipstick trace to ≥1), and severe (ACR >30 mg/mmol or dipstick ≥2) based on each patient’s median value when multiple measurements were made.^11^

We identified CKD, AKI, AKD without AKI, and combinations of these states based on all sCr, eGFR, and albuminuria determinations over the 3 months before and after the index date (eFigure 1 in the Supplement) based on the algorithm provided in the KDIGO Clinical Practice Guideline for AKI^3^ and criteria described by the Acute Disease Quality Initiative consensus statement.^5^ First, the sCr and eGFR on the index date were compared with sCr and eGFR at least 3 months prior to the index date, and all albuminuria measurements prior to the index date were identified for each patient. Patients were classified as having (1) CKD if both the index eGFR and preceding eGFR were less than 60 mL/min/1.73 m^2^ or if albuminuria was present prior to the index date; (2) AKI if the index sCr had increased by more than 0.3 mg/dL (to convert to micromoles per liter, multiply by 88.4) over 2 days or increased or decreased 1.5-fold or more from a nadir value over 7 days within the 3 months preceding the index date; (3) AKD without AKI if the index eGFR was less than 60 mL/min/1.73 m^2^ and the preceding measure was greater than or equal to 60 mL/min/1.73 m^2^ or there was no preceding sCr or eGFR measurement and albuminuria was absent or not measured prior to the index date; and (4) no kidney disease (NKD) if the index and prior eGFR were both greater than or equal to 60 mL/min/1.73 m^2^, albuminuria was absent or not measured, and AKI had not been detected. Second, the sCr, eGFR, and albuminuria measurements on the index date were compared with measurements over the 3 months after the index date. Patients with an increase in sCr greater than or equal to 0.3 mg/dL within a 2-day period or a 1.5-fold or greater increase or decrease in sCr from nadir value within a 7-day interval after the index date were classified as having AKI. For patients not already identified with AKI, those with a decrease in eGFR of 35%, an increase in sCr of 50%, or new onset of albuminuria were classified as having AKD without AKI. Those with AKD based on sCr and eGFR criteria but with an acute care diagnosis known to be associated with AKI (eTable 2 in the Supplement) were classified as having AKI. Patients identified with CKD and AKI in the 3 months before or after the index date were classified as having CKD with AKI. Similarly, those with CKD and AKD without AKI in the 3 months before or after the index date were classified as having CKD with AKD without AKI. The final classification included 6 categories: (1) NKD, (2) AKI, (3) AKD without AKI, (4) CKD, (5) CKD with AKI, and (6) CKD with AKD without AKI.

### Measurement of Covariates

Comorbidities and acute care diagnoses associated with AKI conditions were identified using previously validated and published approaches applied to provincial physician claims, ambulator care, and hospital discharge abstracts.^12,13,14,15,16,17^ Measures of health care system contacts before and after the index date included nephrologist visits, hospitalizations, and sCr measurements identified from physician claims, hospital discharge abstracts, and provincial laboratory databases.^7^

### Ascertainment of Study Outcomes

Participants were followed up for 8.5 years (study end date, June 30, 2016) from the index date for the study outcomes of time to death of any cause, development of CKD, progression of CKD, and ESKD with initiation of kidney replacement therapy. Mortality was identified from Alberta provincial vital statistics records. For the primary analysis, development of CKD was defined as sustained eGFR less than 60 mL/min/1.73 m^2^ or albuminuria (ACR >3 mg/mmol) on a least 2 measurements separated by more than 3 months among those without preexisting CKD (baseline eGFR ≥60 mL/min/1.73 m^2^ and no albuminuria). Progression of CKD was defined as a decline of 35% or greater in eGFR from baseline more than 3 months after the index date among those with preexisting CKD (baseline eGFR <60 mL/min/1.73 m^2^ or albuminuria).^18^ We identified ESKD with initiation of kidney replacement therapy using records for long-term dialysis or kidney transplantation from the Northern and Southern Alberta Renal Programs.

### Statistical Analysis

We compared baseline characteristics according to the NKD, CKD, AKI, and AKD states using analysis of variance for continuous variables and χ^2^ tests for categorical variables. We determined the proportion of patients with each condition based on the 2 ascertainment periods around the index date as functions of the number of adult residents (aged >18 years) of Alberta with an sCr measurement in 2008, and the estimated adult Alberta population based on 2008 provincial census data.

Time from index date to each of the clinical outcomes, emigration from the province, or the study end date was determined and plotted using cumulative incidence curves. Outcomes of patients with CKD, AKI, AKD without AKI, and combinations of these states were examined relative to patients with NKD using Cox proportional hazards regression for time to all-cause mortality regardless of intermediate events. To avoid overestimating the incidence of nonfatal outcomes that can result from censoring for death using Cox regression, we used Fine and Gray^19^ competing risk regression to model death as a competing risk and obtain sub–hazard ratios for the outcomes of CKD development, CKD progression, and ESKD. Participants with preexisting CKD were excluded from the analyses for development of CKD, and patients without preexisting CKD were excluded from the analyses for progression of CKD. We fit unadjusted models, as well as models with stepwise progressive adjustment for age and sex only and adjusted for age, sex, social assistance, and individual comorbidities. We determined that the proportional hazard assumption for between-group comparisons with NKD was satisfied by using graphical methods and testing time-dependent effects. We compared the impact of adding AKD identification to improve discrimination for clinical outcomes based on the integrated discrimination improvement, and to reclassify patients into higher- or lower-risk categories based on risk thresholds of less than 1%, 1% to 4.9%, 5% to 9.9%, 10% to 19.9%, and greater than or equal to 20% using the continuous net reclassification index.^20^ Our threshold for statistical significance was set at 2-sided *P* < .05.

We performed a sensitivity analysis based on an alternative definition for AKD requiring an index eGFR less than 60 mL/min/1.73 m^2^ with a decline greater than 10 mL/min/60 mL/min/1.73 m^2^ from previous baseline or subsequent increase in sCr by 50% within 7 days to 3 months. We also conducted 2 additional subgroup analyses to avoid potential misclassification of unrecognized CKD as AKD; the first restricted the cohort to those with prior eGFR greater than 60 mL/min/1.73 m^2^ (excluding patients with no prior sCr measurements). The second was restricted to patients without albuminuria at the index date (excluding patients with unmeasured, moderate, or severe albuminuria).

## Results

### Identification of CKD, AKI, and AKD Without AKI

There were 1 109 099 eligible adult residents without ESKD with an index sCr measurement in Alberta in 2008 (eFigure 2 in the Supplement). The mean (SD) age was 52.3 (17.6) years, and 43.0% were male. A total of 921 116 individuals (83.0%) were identified with NKD, 118 397 (10.7%) with CKD, 15 777 (1.4%) with AKI, 42 487 (3.8%) with AKD without AKI, 5015 (0.4%) with CKD with AKI, and 6307 (0.6%) with CKD with AKD without AKI. The number of patients with CKD, AKI, and AKD identified by applying each component of the KDIGO criteria is shown in Table 1, with further details of the frequency of each criterion for AKD in eFigure 3 in the Supplement. From the total cohort, 797 594 individuals (71.9%) were identified with preceding eGFR greater than 60 mL/min/1.73 m^2^, while 575 263 (51.9%) were identified without preexisting albuminuria.

**Table 1.** Participants With CKD, AKI, and AKD Based on Stepwise Application of Each of the Criterion Used for Identification

Category and Criteria	No. (%)
No kidney disease	
Total	921 116 (100)
CKD^a^	
Prior eGFR <60 mL/min/1.73 m^2^	66 955 (51.6)
Preexisting albuminuria^b^	62 764 (48.4)
Identified by albumin to creatinine ratio	17 331 (27.6)
Identified by dipstick	45 433 (72.4)
Total	129 719 (100)
AKI^a^	
Increase in sCr >0.3 mg/dL in 2 d or >50% in 7 d	8807 (42.4)
Decrease in sCr >50% in 7 d	8906 (42.8)
Coincident acute care diagnosis associated with AKI	3079 (14.8)
Total	20 792 (100)
AKD without AKI^a^^,^^c^	
Prior eGFR >60 mL/min/1.73 m^2^, index eGFR <60 mL/min/1.73 m^2^	23 049 (47.2)
No prior eGFR measure, index eGFR <60 mL/min/1.73 m^2^	4903 (10.0)
Increase sCr >50% in >7 d for <90 d	3825 (7.8)
Decrease in eGFR >35% in >7 d for <90 d	1019 (2.1)
Development of albuminuria^d^	15 998 (32.8)
Identified by albumin to creatinine ratio	4221 (26.4)
Identified by dipstick	11 777(73.6)
Total	48 794 (100)

Abbreviations: AKD, acute kidney diseases and disorders; AKI, acute kidney injury; CKD, chronic kidney disease; eGFR, estimated glomerular filtration rate; sCr, serum creatinine.

SI conversion factor: To convert creatinine to μmol/L, multiply by 88.4.

^a^ The number of participants with CKD is not mutually exclusive with numbers of participants with AKI and AKD.

^b^ Albuminuria was identified by urine albumin to creatinine ratio greater than or equal to 3 mg/mmol or positive dipstick urinalysis.

^c^ Among participants with AKD, 33 097 (77.9%) had a decrease in eGFR greater than 10 mL/min/1.73 m^2^.

^d^ Among 15 998 participants with AKD identified by albuminuria, 5071 (31.7%) had a preceding urine dipstick or albumin to creatinine ratio measurement that was normal.

### Incidence of CKD, AKI, and AKD Without AKI

**Figure 1.**
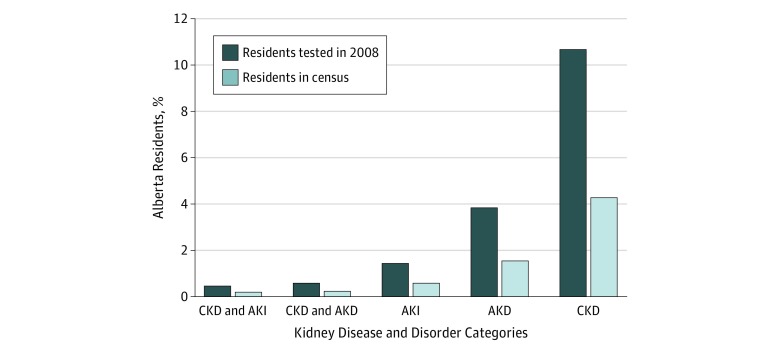
Percentage of Adult Residents of Alberta, Canada, With Chronic Kidney Disease (CKD), Acute Kidney Injury (AKI), Acute Kidney Diseases and Disorders (AKD), and Combinations of These Conditions in 2008 Conditions were identified by testing for serum creatinine, estimated glomerular function rate, and albuminuria.

The percentages of tested participants with CKD, AKI, AKD without AKI, and combinations of these conditions in Alberta in 2008, and the frequency based on Alberta provincial census data, are shown in Figure 1. The incidence of AKD without AKI was 3.8 per 100 adults tested, making it less common than CKD (10.6 per 100 adults tested) but more common than AKI (1.4 per 100 adults tested). An additional 0.6 per 100 adults tested were identified who had CKD with AKD without AKI.

### Characterization of CKD, AKI, and AKD Without AKI

Compared with those with NKD, patients with AKD without AKI were older, had lower eGFR, and had more comorbidities than those with NKD (Table 2). In all, 1.6% of those with AKD without AKI and 7.4% of those with CKD with AKD without AKI had seen a nephrologist in the year prior to their index measurement. Among the 15 777 patients with AKI, 10 710 (67.9%) were detected in an inpatient or emergency department setting, whereas among 42 287 participants with AKD, 32 694 (77.0%) were identified upon outpatient testing. Following detection, 1.6% of participants with AKD without AKI and 7.5% of participants with CKD with AKD without AKI saw a nephrologist within 3 months.

**Table 2.** Baseline Characteristics of 1 109 099 Residents of Alberta, Canada, Who Received Serum Creatinine Testing in 2008, According to CKD, AKI, AKD, and Combinations of These States

Variable	NKD (n = 921 116)	CKD (n = 118 397)	AKD Without AKI (n = 42 487)	AKI (n = 15 777)	CKD With AKD Without AKI (n = 6307)	CKD With AKI (n = 5015)	*P* Value
Sociodemographic factors^a^							
Age, mean (SD), y	50.0 (16.3)	65.4 (18.7)	63.7 (17.3)	62.5 (18.9)	72.4 (14.1)	74.9 (14.9)	<.001
Male, No. (%)	392 620 (42.6)	53 109 (44.9)	18 029 (42.4)	8035 (50.9)	3131 (49.6)	2387 (47.6)	<.001
Socioeconomic status, No. (%)							
Social beneficiaries program	250 065 (27.2)	73 823 (62.4)	24 901 (58.6)	8453 (53.6)	4754 (75.4)	4022 (80.2)	<.001
Health premium subsidy	33 964 (3.7)	3468 (2.9)	1875 (4.4)	1375 (8.7)	207 (3.3)	215 (4.3)	
No premium subsidy	614 330 (66.7)	38 444 (32.5)	14 539 (34.2)	5091 (32.3)	1186 (18.8)	638 (12.7)	
Kidney laboratory tests							
Index eGFR, mean (SD), mL/min/1.73 m^2^^b^	92.6 (17.8)	66.8 (26.4)	67.3 (23.1)	70.6 (27.6)	49.5 (15.1)	46.3 (22.5)	<.001
Albuminuria, No. (%)							
None	533 401 (57.9)	24 850 (21.0)	12 882 (30.3)	3447 (21.8)	144 (2.3)	539 (10.8)	<.001
Moderate	0	55 970 (47.3)	12 131 (28.6)	1021 (6.5)	4575 (72.5)	1804 (36.0)	
Severe	0	10 163 (8.6)	1493 (3.5)	223 (1.4)	1281 (20.3)	837 (16.7)	
Unmeasured	387 715 (42.1)	27 414 (23.2)	15 981 (37.6)	11 086 (70.3)	307 (4.9)	1835 (36.6)	
Comorbidities, No. (%)							
Cancer	23 447 (2.6)	5895 (5.0)	2484 (5.8)	1597 (10.1)	442 (7.0)	526 (10.5)	<.001
Myocardial infarction	14 402 (1.6)	5436 (4.6)	1831 (4.3)	1774 (11.2)	414 (6.6)	725 (14.5)	<.001
Congestive heart failure	8025 (0.9)	5209 4.4)	1553 (3.7)	1632 (10.3)	478 (7.6)	857 (17.1)	<.001
Cerebrovascular disease	12 105 (1.3)	4477 (3.8)	1635 (3.8)	1126 (7.1)	374 (5.9)	483 (9.6)	<.001
Peripheral vascular disease	4563 (0.5)	2370 (2.0)	733 (1.7)	621 (3.9)	217 (3.4)	254 (5.1)	<.001
Chronic pulmonary disease	58 987 (6.4)	11 075 (9.4)	4378 (10.3)	2766 (17.5)	728 (11.5)	970 (19.3)	<.001
Dementia	7270 (0.8)	4432 (3.7)	1349 (3.2)	992 (6.3)	366 (5.8)	474 (9.4)	<.001
Diabetes	26 102 (2.8)	6338 (5.4)	2035 (4.8)	930 (5.9)	388 (6.2)	291 (5.8)	<.001
Metastatic carcinoma	1225 (0.1)	288 (0.2)	189 (0.4)	187 (1.2)	29 (0.5)	41 (0.8)	<.001
Liver disease (mild)	3846 (0.4)	531 (0.4)	300 (0.7)	328 (2.1)	44 (0.7)	80 (1.6)	<.001
Liver disease (moderate or severe)	757 (0.1)	113 (0.1)	91 (0.2)	254 (1.6)	22 (0.4)	26 (0.5)	<.001
Peptic ulcer disease	3998 (0.4)	766 (0.6)	270 (0.6)	191 (1.2)	50 (0.8)	50 (1)	<.001
Rheumatic disease	8648 (0.9)	1532 (1.3)	689 (1.6)	339 (2.2)	105 (1.7)	99 (2.0)	<.001
Paraplegia and hemiplegia	1039 (0.1)	170 (0.1)	91 (0.2)	79 (0.5)	7 (0.1)	20 (0.4)	<.001
Hypertension	64 155 (7.0)	10 557 (8.9)	4400 (10.4)	1560 (9.9)	633 (10.0)	468 (9.3)	<.001
Processes of care							
Hospitalizations in preceding y, median (IQR), No.	0 (0-0)	0 (0-0)	0 (0-0)	0 (0-1)	0 (0-0)	0 (0-1)	<.001
Serum creatinine measures prior to index measurement, median (IQR), No.	3 (1-5)	6 (2-12)	5 (2-10)	7 (2-21)	8 (3-16)	13 (6-26)	<.001
Serum creatinine measures in y prior to index measurement, median (IQR), No.	0 (0-1)	1 (0-2)	0 (0-1)	1 (0-6)	1 (0-2)	2 (0-6)	<.001
Nephrologist visit in y prior to index serum creatinine measure, No. (%)	3871 (0.4)	5334 (4.5)	698 (1.6)	758 (4.8)	469 (7.4)	467 (9.3)	<.001
Location of testing at identification, No. (%)							
Outpatient	794 037 (86.2)	107 014 (90.4)	32 694 (76.9)	5067 (32.1)	5443 (86.3)	2278 (45.4)	<.001
Inpatient	22 127 (2.4)	3467 (2.9)	2211 (5.2)	3807 (24.1)	217 (3.4)	883 (17.6)	
Emergency department	104 952 (11.4)	7916 (6.7)	7582 (17.8)	6903 (43.7)	647 (10.3)	1854 (37.0)	

Abbreviations: AKD, acute kidney diseases and disorders; AKI, acute kidney injury; CKD, chronic kidney disease; eGFR, estimated glomerular filtration rate; IQR, interquartile range; NKD, no kidney disease.

^a^ Information on ethnicity of the cohort not available.

^b^ Calculated using the Chronic Kidney Disease Epidemiology Collaboration equation.

### Risk of Subsequent Clinical Outcomes by CKD, AKI, and AKD Without AKI Status

**Figure 2.**
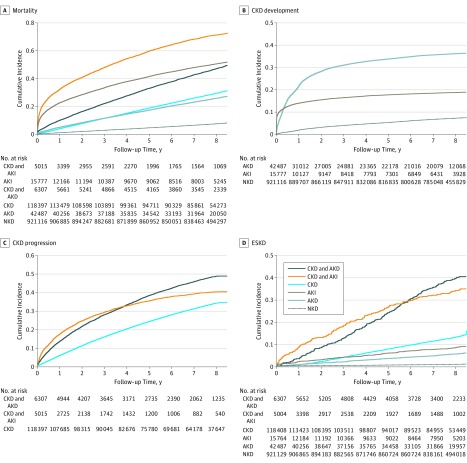
Cumulative Incidence Curves for Mortality, Incident Chronic Kidney Disease (CKD), Progressive CKD, and End-Stage Kidney Disease (ESKD), According to Kidney Disease Classification of Residents of Alberta, Canada, Who Received Serum Creatinine Testing in 2008 The cumulative incidence curves of mortality were plotted using the Kaplan-Meier function. The curves for other outcomes were plotted using the cumulative incidence function. Note that the y-axis scale varies by outcome. AKD indicates acute kidney diseases and disorders; AKI, acute kidney injury; and NKD, no kidney disease.

The time from index sCr measurement to death, development of CKD (for those without preexisting CKD), progression of CKD (for those with preexisting CKD), and ESKD treated with kidney replacement therapy is shown in Figure 2. Over a median (interquartile range) of 6.0 (5.7-6.3) years of follow-up, the cumulative incidence of all 4 of these outcomes was greater for patients with AKD without AKI and CKD with AKD without AKI compared with patients with no kidney disease. The associations with clinical outcomes remained significantly greater for patients with AKD without AKI and CKD with AKD without AKI than patients without kidney disease in models adjusted for age and sex as well as in multivariable models further adjusted for socioeconomic status and comorbidities (Table 3 and eTable 3 in the Supplement). Compared with those with NKD, the risk of all-cause death was significantly higher for those with AKD without AKI (25.8% vs 7.3%; adjusted hazard ratio [HR], 1.42; 95% CI, 1.39-1.45) and CKD with AKD without AKI (47.2% vs 7.3%; adjusted HR, 1.92; 95% CI, 1.85-2.00). The incidence of ESKD was low; however, risk was higher for those with AKD without AKI (0.6% vs 0.1%; adjusted sHR, 8.56; 95% CI, 7.32-10.01) and CKD with AKD without AKI (4.1% vs 0.1%; adjusted HR, 65.95; 95% CI, 55.91-77.80) than for those with NKD. Results showed that AKD without AKI was also associated with higher risks of developing new CKD (37.4% vs 7.4%%; adjusted sHR, 3.17; 95% CI, 3.10-3.23) and progression of preexisting CKD (49.5% vs 34.6%; adjusted sHR, 1.38; 95% CI, 1.33-1.44). Associations between AKD without AKI and clinical outcomes were similar for patients with prior eGFR greater than 60 mL/min/1.73 m^2^ as well as those without prior albuminuria (eTable 4 and eTable 5 in the Supplement) and when varying components of AKD criteria were examined (eTable 6 in the Supplement).

**Table 3.** Unadjusted and Adjusted Outcomes of Residents of Alberta, Canada, Who Received Serum Creatinine Testing in 2008, According to CKD, AKI, AKD Without AKI, and Combinations of These States

Outcome	Events, No. (%)	Total Follow-up, y	Hazard Ratio^a^ (95% CI)		
			Crude^a^	Age- and Sex-Adjusted^a^	Multivariable-Adjusted^a^^,^^b^
Mortality (n = 1 109 099)					
NKD	67 655 (7.3)	7 029 114	1 [Reference]	1 [Reference]	1 [Reference]
CKD with AKD without AKI	2977 (47.2)	37 000	8.33 (8.03-8.65)	1.49 (1.46-1.52)	1.92 (1.85-2.00)
CKD with AKI	3585 (71.5)	19 574	18.75 (18.13-19.39)	4.47 (4.36-4.57)	3.30 (3.19-3.41)
AKD without AKI	10 940 (25.8)	291 157	3.90 (3.82-3.98)	2.08 (2.00-2.15)	1.42 (1.39-1.45)
AKI	7964 (50.5)	80 864	10.15 (9.91-10.39)	4.20 (4.06-4.34)	3.23 (3.16-3.31)
CKD	34 816 (29.4)	804 081	4.50 (4.44-4.55)	1.42 (1.4-1.44)	1.37 (1.35-1.39)
CKD development for those without CKD^c^ (n = 979 380)					
NKD	68 355 (7.4)	6 724 569	1 [Reference]	1 [Reference]	1 [Reference]
AKD without AKI	15 884 (37.4)	199 813	6.54 (6.43-6.66)	3.23 (3.16-3.30)	3.17 (3.1-3.23)
AKI	3066 (19.4)	65 813	2.98 (2.87-3.10)	1.43 (1.37-1.49)	1.32 (1.26-1.38)
CKD progression for those with CKD (n = 129 719)					
CKD	40 907 (34.6)	680 887	1 [Reference]	1 [Reference]	1 [Reference]
CKD with AKD without AKI	3123 (49.5)	27 205	1.67 (1.61-1.74)	1.42 (1.36-1.47)	1.38 (1.33-1.44)
CKD with AKI	2064 (41.2)	13 488	1.38 (1.31-1.45)	1.37 (1.32-1.42)	1.38 (1.33-1.44)
ESKD (n = 1 109 099)					
NKD	653 (0.1)	7 027 508	1 [Reference]	1 [Reference]	1 [Reference]
CKD with AKD without AKI	256 (4.1)	36 351	57.82 (50.03-66.81)	74.94 (63.67-88.21)	65.95 (55.91-77.80)
CKD with AKI	176 (3.5)	19 195	50.11 (42.39-59.23)	68.22 (56.71-82.06)	52.20 (43.20-63.08)
AKD without AKI	236 (0.6)	290 730	7.79 (6.71-9.04)	9.39 (8.04-10.97)	8.56 (7.32-10.01)
AKI	135 (0.9)	80 752	12.00 (9.97-14.44)	13.24 (10.95-16.01)	9.89 (8.12-12.03)
CKD	1590 (1.3)	799 734	18.88 (17.24-20.68)	22.65 (20.48-25.05)	21.54 (19.44-23.87)

Abbreviations: AKD, acute kidney diseases and disorders; AKI, acute kidney injury; CKD, chronic kidney disease; ESKD, end-stage kidney disease; NKD, no kidney disease.

^a^ Hazard ratios were determined using Cox proportional hazards models for mortality, and sub–hazard ratios were determined using Fine and Gray competing risk models for CKD development, CKD progression, and ESKD.

^b^ Full multivariable adjusted models adjusted for age, sex, socioeconomic status, Aboriginal ethnicity, individual Charlson comorbidities, and hypertension.

^c^ A total of 28 835 cases (33.0%) of individuals developing CKD were based on sustained albuminuria and 58 470 (77.0%) were based on a sustained reduced estimated glomerular filtration rate.

### Additional Prognostic Value of Identification of AKD

Identifying patients with AKD without AKI and CKD with AKD without AKI (who otherwise would have been classified with NKD and CKD, respectively) improved the ability to correctly classify patients for risk of all outcomes (eTable 7 in the Supplement). For the outcomes of mortality, CKD progression, and ESKD, additive net reclassification improvement was achieved by improved classification of patients who experienced events into higher-risk categories, whereas for the outcome of CKD developed, improved additive net reclassification was achieved by improved classification of patients who did not develop CKD into lower-risk categories.

## Discussion

In this large cohort study of more than 1 million people with sCr and albuminuria measurements, we examined the incidence and outcomes of AKD without AKI in relation to CKD, AKI, and combinations of these disorders. We found that AKD without AKI was common and, similar to CKD and AKI, was associated with increased risks of death, frequent development or progression of CKD, and less frequent but increased risk of progression to ESKD compared with patients with NKD over 6 years following identification. The highest risks of death, progression of CKD, and ESKD were observed among patients with AKD without AKI in combination with CKD. Adding AKD without AKI to the classification of kidney disorders improved identification of patients who died or developed new onset or progressive CKD or ESKD. These findings illustrate that AKD without AKI is frequently observed, identifies patients not recognized by current AKI and CKD criteria, and is associated with overall modestly increased risk of long-term adverse outcomes.

Although AKD criteria were first published in the KDIGO clinical practice guideline for AKI in 2012, there is limited information on the causes and clinical characteristics of patients who develop AKD without AKI.^21^ In principle, AKD without AKI might be caused by episodes of decreased kidney perfusion or parenchymal kidney diseases in which the decrement in GFR is too small or evolves too slowly to raise the sCr within the time limit of the AKI criteria, or to be detected with infrequent sCr measurements. Criteria for AKD have been applied previously in case series of patients who underwent kidney biopsy during hospitalization.^22^ Acute tubular necrosis was almost twice as common among patients with AKI as it was among those with AKD without AKI, while acute tubulointerstitial nephritis was more common among patients with AKD without AKI than among those with AKI. Crescentic glomerulonephritis and thrombotic microangiopathy were also observed in both AKI and AKD. Patients with biopsy-proven crescentic glomerulonephritis also presented with criteria for AKD without AKI in more than 10% of cases in another case series.^23^ These findings provide some insight into the nature of underlying kidney diseases that may fall within the spectrum of AKD and address points that have been emphasized in commentaries from the National Kidney Foundation Kidney Diseases Quality Initiative^24^ and Canadian Society of Nephrology,^25^ including recognition of the broad range of kidney diseases that may fall within AKD criteria and the need for clinical recognition and investigation to distinguish the array of underlying causes of disease and provide prompt treatment. However, the pathology identified in association with AKD in these reports is likely biased by selection of patients requiring a kidney biopsy. Our study provides novel information on the relatively high incidence of AKD, which suggests that most cases are unlikely to be attributable to glomerulonephritis or interstitial nephritis and highlights the prognostic significance of this presentation of kidney disease that is distinct from the criteria for AKI and CKD.

### Limitations

There are limitations to our study. First, our study does not characterize clinical features, including underlying causes and treatments for patients who met criteria for AKD, and thus does not identify causes of kidney disease or resolve how patients with AKD should be cared for in clinical practice. However, our findings do provide epidemiological insight into the incidence and prognostic relevance of AKD that can help inform whether changes to the classification systems for kidney diseases proposed in the KDIGO Clinical Practice Guideline for AKI should incorporate criteria for AKD within approaches to surveillance and detection of kidney disease in clinical care. Second, we focused on the use of sCr, eGFR, and albuminuria criteria for identifying CKD, AKI, and AKD without AKI in this study in keeping with the classification scheme proposed in the KDIGO guideline. Serum creatinine is frequently measured in clinical practice but may be influenced by non-GFR determinants, including age, sex, race, body composition, inflammation, diet, and medications. Relatively small changes in eGFR from a baseline eGFR greater than 60 mL/min/1.73 m^2^ resulting from physiological and measurement variability may contribute to identification of many cases of AKD based on the current KDIGO criteria. Albuminuria quantification may vary between measurements and was less frequently measured in our cohort, which could also lead to misclassification of CKD vs AKD and underestimate the prevalence of CKD while overestimating the prevalence of AKD. Hematuria, pyuria, other abnormalities of urine sediment, and imaging findings were not ascertained but are also important criteria for identification of kidney diseases.^5,21^ Like CKD and AKI, there may be a variety of causes of AKD, and not all reductions in eGFR may be associated with poor outcomes or require the same investigations or management. For example, some interventions, such as antihypertensive medication use for intensive blood pressure lowering, may reduce kidney function but improve outcomes.^26,27^ Third, 10% of patients in our study who met criteria for AKD did not have a preceding sCr measurement, so it is possible that some patients classified with AKD truly had AKI or CKD but did not have sufficient repeated sCr measurements to meet the diagnostic criteria for these conditions at time of testing. Patients with AKI and AKD were more likely to be hospitalized and have follow-up sCr and eGFR testing, which may have increased the opportunity to detect incident or progressive CKD in this cohort. However, our results reflect how abnormal kidney function is recognized in real-world care and provide comprehensive information for patients from a geographic region with universal access to health care. Fourth, the study was conducted in a Canadian province, and results may not be generalizable to populations with different ethnic distributions or determinants of access to care and laboratory testing.

## Conclusions

We found that AKD without AKI was common among patients in a universal health system who underwent sCr and albuminuria testing and, like CKD and AKI, AKD was associated with risk of adverse outcomes. These findings suggest that the incorporation of AKD into clinical and research initiatives for kidney disease would increase recognition of patients at risk of adverse outcomes who are not identified by current AKI and CKD criteria. However, the clinical characteristics of AKD remain to be determined, and further research is needed to characterize the clinical causes of AKD, identify their potential varying associations with outcomes, and understand how clinicians should care for people with AKD.

## Notes

Supplement. **eTable 1.** Criteria for Kidney Disease Improving Global Outcomes Definitions of Kidney Diseases and Disorders **eTable 2.** Most Responsible Diagnosis Codes Used to Identify Coincident Acute Care Diagnoses Associated With AKI **eTable 3.** Hazard Ratios From Multivariable Cox Proportional Hazards Model for Mortality and Subhazard Ratios From Fine and Gray Competing Risk Models for Nonfatal Outcomes for Demographic Characteristics, Comorbidities, and CKD, AKI, AKD Without AKI and Combinations of These States, Among Patients Who Received Scr Testing in 2008 **eTable 4.** Unadjusted and Adjusted Outcomes Restricted to Alberta Residents With eGFR > 60 mL/min/1.73m^2^ Prior to the Index Dates (Excludes Patients With No Prior Scr Measurements) Who Received Scr Testing in 2008, According to CKD, AKI, AKD Without AKI, and Combinations of These States **eTable 5.** Unadjusted and Adjusted Outcomes Restricted to Alberta Residents Without Albuminuria Prior to the Index Dates (Excludes Patients With Unmeasured, Moderate, or Severe Albuminuria) Who Received Scr Testing in 2008, According to CKD, AKI, AKD Without AKI, and Combinations of These States **eTable 6.** Unadjusted and Adjusted Outcomes for Alberta Residents Who Received Scr Testing in 2008, Using an Alternate Definition for AKD Requiring an Index eGFR < 60 mL/min/1.73m^2^ With a Decline > 10 mL/min/1.73m^2^ From Previous Baseline or Subsequent Increase in Scr by 50% Within 7 Days to 3 Months **eTable 7.** Categorical Net Reclassification Improvement (NRI) and Integrated Discrimination Improvement (IDI) With Addition of AKD Criteria to Kidney Disease Classification for Alberta Residents Who Received Scr Testing in 2008, According to CKD, AKI, AKD, and Combinations of These States **eFigure 1.** Time Frames and Sequence of Steps for Using Serum Creatinine / Estimated Glomerular Filtration Rate and Albuminuria Determinations to Characterize NKD, CKD, AKD, AKI **eFigure 2.** Study Cohort Formation **eFigure 3.** Numbers of Participants With AKD Based on Meeting One of More of Each of the Criterion Used for AKD Identification
