# Supplementary material for: Incidence and Prognosis of Acute Kidney Diseases and Disorders Using an Integrated Approach to Laboratory Measurements in a Universal Health Care System
Source: JAMA Netw Open. 2019 Apr 5;2(4):e191795. doi: 10.1001/jamanetworkopen.2019.1795 (PMC6450331; doi:10.1001/jamanetworkopen.2019.1795)
Supplement: Supplement. — eTable 1. Criteria for Kidney Disease Improving Global Outcomes Definitions of Kidney Diseases and Disorders eTable 2. Most Responsible Diagnosis Codes Used to Identify Coincident Acute Care Diagnoses Associated With AKI eTable 3. Hazard Ratios From Multivariable Cox Proportional Hazards Model for Mortality and Subhazard Ratios From Fine and Gray Competing Risk Models for Nonfatal Outcomes for Demographic Characteristics, Comorbidities, and CKD, AKI, AKD Without AKI and Combinations of These States, Among Patients Who Received Scr Testing in 2008 eTable 4. Unadjusted and Adjusted Outcomes Restricted to Alberta Residents With eGFR > 60 mL/min/1.73m2 Prior to the Index Dates (Excludes Patients With No Prior Scr Measurements) Who Received Scr Testing in 2008, According to CKD, AKI, AKD Without AKI, and Combinations of These States eTable 5. Unadjusted and Adjusted Outcomes Restricted to Alberta Residents Without Albuminuria Prior to the Index Dates (Excludes Patients With Unmeasured, Moderate, or Severe Albuminuria) Who Received Scr Testing in 2008, According to CKD, AKI, AKD Without AKI, and Combinations of These States eTable 6. Unadjusted and Adjusted Outcomes for Alberta Residents Who Received Scr Testing in 2008, Using an Alternate Definition for AKD Requiring an Index eGFR < 60 mL/min/1.73m2 With a Decline > 10 mL/min/1.73m2 From Previous Baseline or Subsequent Increase in Scr by 50% Within 7 Days to 3 Months eTable 7. Categorical Net Reclassification Improvement (NRI) and Integrated Discrimination Improvement (IDI) With Addition of AKD Criteria to Kidney Disease Classification for Alberta Residents Who Received Scr Testing in 2008, According to CKD, AKI, AKD, and Combinations of These States eFigure 1. Time Frames and Sequence of Steps for Using Serum Creatinine / Estimated Glomerular Filtration Rate and Albuminuria Determinations to Characterize NKD, CKD, AKD, AKI eFigure 2. Study Cohort Formation eFigure 3. Numbers of Participants With AKD Based on Meeting One [file jamanetwopen-2-e191795-s001.pdf]

Supplementary Online Content

James MT, Levey AS, Tonelli M, et al. Incidence and prognosis of acute kidney diseases and disorders using an integrated approach to laboratory measurements in a universal health care system. *JAMA Netw Open*. 2019;2(4):e191795. doi:10.1001/jamanetworkopen.2019.1795

eTable 1. Criteria for Kidney Disease Improving Global Outcomes Definitions of Kidney Diseases and Disorders

eTable 2. Most Responsible Diagnosis Codes Used to Identify Coincident Acute Care Diagnoses Associated With AKI

eTable 3. Hazard Ratios From Multivariable Cox Proportional Hazards Model for Mortality and Subhazard Ratios From Fine and Grey Competing Risk Models for Nonfatal Outcomes for Demographic Characteristics, Comorbidities, and CKD, AKI, AKD Without AKI and Combinations of These States, Among Patients Who Received Scr Testing in 2008

eTable 4. Unadjusted and Adjusted Outcomes Restricted to Alberta Residents With eGFR > 60 mL/min/1.73m^2 Prior to the Index Dates (Excludes Patients With No Prior Scr Measurements) Who Received Scr Testing in 2008, According to CKD, AKI, AKD Without AKI, and Combinations of These States

eTable 5. Unadjusted and Adjusted Outcomes Restricted to Alberta Residents Without Albuminuria Prior to the Index Dates (Excludes Patients With Unmeasured, Moderate, or Severe Albuminuria) Who Received Scr Testing in 2008, According to CKD, AKI, AKD Without AKI, and Combinations of These States

eTable 6. Unadjusted and Adjusted Outcomes for Alberta Residents Who Received Scr Testing in 2008, Using an Alternate Definition for AKD Requiring an Index eGFR < 60 mL/min/1.73m^2 With a Decline > 10 mL/min/1.73m^2 From Previous Baseline or Subsequent Increase in Scr by 50% Within 7 Days to 3 Months

eTable 7. Categorical Net Reclassification Improvement (NRI) and Integrated Discrimination Improvement (IDI) With Addition of AKD Criteria to Kidney Disease Classification for Alberta Residents Who Received Scr Testing in 2008, According to CKD, AKI, AKD, and Combinations of These States

eFigure 1. Time Frames and Sequence of Steps for Using Serum Creatinine / Estimated Glomerular Filtration Rate and Albuminuria Determinations to Characterize NKD, CKD, AKD, AKI

eFigure 2. Study Cohort Formation

© 2019 James MT et al. *JAMA Network Open.*

eFigure 3. Numbers of Participants With AKD Based on Meeting One of More of Each of the Criterion Used for AKD Identification

This supplementary material has been provided by the authors to give readers additional information about their work.

© 2019 James MT et al. *JAMA Network Open.*

eTable 1: Criteria for Kidney Disease Improving Global Outcomes Definitions of Kidney Diseases and Disorders

**Criteria**	**AKI**	**AKD**	**CKD**	**NKD***
Duration	Within 7 days	≤3 months	>3 months	
Function Alteration	Oliguria for ≥6 hours, or<br>Increase in Scr by ≥0.3 mg/dl in 48 hours, or<br>Increase in Scr by ≥50% in 7 days	AKI, or<br>GFR <60 ml/min/1.73 m^2, or<br>Decrease in GFR by ≥35%, OR<br>Increase in Scr by ≥50%	GFR <60 ml/min/1.73 m^2	GFR ≥60 ml/min/1.73 m^2, and<br>Stable Scr
Structural Alteration	Not defined	Marker of kidney damage (albuminuria)	Marker of kidney damage (albuminuria)	No marker of kidney damage

Abbreviations: AKI = Acute Kidney Injury, AKD = Acute Kidney Diseases and Disorders, CKD = Chronic Kidney Disease, GFR = Glomerular Filtration Rate, NKD = No Kidney Disease, Scr = Serum creatinine

*Implies no functional or structural criteria according to the definitions for AKI, AKD or CKD.

© 2019 James MT et al. *JAMA Network Open.*

eTable 2: Most responsible diagnosis codes used to identify coincident acute care diagnoses associated with AKI

Diagnosis*	ICD Codes	Number (%) of Additional Participants Identified with AKI using Codes
Sepsis	ICD-9-CM 038.x, 112.5, 112.81, 02.02, 79.07, 78.559, ICD-10-CA A02.1, A22.7, A24.1, A26.7, A32.7, A40.0, A40.1, A40.2, A40.3, A40.8, A40.9, A41, A41.0, A41.1., A41.2, A41.3, A41.4, A41.5, A41.50, A41.51, A41.52, A41.58, A41.8, A41.80, A41.88, A41.9, A42.7, B37.7, B37.6, A01.0	38 (1.2)
Pneumonia	ICD-9-CM 460.x, 481, 482.x, 483.x, 484.x, 485, 486, ICD-10 J10.0, J11.0, J11.1, J12.9, J13, J14, J15.x, J16.8, J17.0, J17.2, J17.3, J17.8, J18.0, J18.1, J18.8, and J18.9	284 (9.2)
Acute myocardial infarction	ICD-9-CM 410.x, 412, ICD-10 I21, I22, I25.2	101 (3.3)
Heart failure	ICD-9-CM 428.x, ICD-10 I50.x	146 (4.7)
Gastrointestinal hemorrhage	ICD-9-CM 578.x, ICD-10-CA K92.0, K92.1,K92.2	82 (2.7)
Acute pancreatitis	ICD-9-CM 577.0, ICD-10-CA K85.9, K85.0, K85.1, K85.2, K85.3, K85.8, K85.9	34 (1.1)
Liver failure	ICD-9-CM 570, ICD-10-CA K72, K72.0, K72.1, K72.9	17 (0.6)
Rhabdomyolysis	ICD-9-CM 728.88, ICD-10-CA M62.82, T79.6	18 (0.6)
Cellulitis	ICD 9-CM 682.9, ICD-10 L03.9, L03.91	158 (5.1)
Urinary Tract Infection	ICD-9-CM 599.0, ICD-10 N39.0	822 (26.7)
Diarrhea or enteritis	ICD-9CM 787.91, 008.8 ICD-10 K52.2, K52.89, R19.7, A08.8	616 (20.0)
Nausea and vomiting	ICD-9-CM 787.01, ICD-10 diagnosis codes R11.x	237 (7.7)
Dehydration	ICD-9-CM 276.51, ICD-10 diagnosis code E86.0	363 (11.8)
Acute tubular necrosis	ICD-9-CM diagnosis codes 584, 584.5, 584.6, 584.7, 584.8, 584.9, ICD-10 diagnosis codes N17, N17.0, N17.1, N17.2, 17.8, N17.9	163 (5.3)
Major surgery or interventional procedures	ICD-9-CM Procedure codes 36.1x, 36.2x, 38.34, 38.64, 38.44, 39.71, 93.90, 93.92, 96.01, 96.04, 96.05, 96.70, 96.71, 96.72, 36.01, 36.02. 36.05, 36.06<br>ICD-10 CA/CCI 1IJ76,1HU90, 1HU80, 1HV90, 1HV80, 1HT90, 1HT80, 1HS90, 1HS80, 1HW, I71.4, 1.KA.80.LA-XXN or 1.KA.80.GQ-NRN, or CCI codes 1.KA.76.MZ-XXN or 1.KA.76.NB-XXN, 1.KA.50.GQ-OA (GQ-BD/GS-BD), 1.KE.50.GQ-OA (GQ-BD/GS-BD), 1.GZ.31, 31P10, 1IJ50, 1IJ5GQ,1IJ57GQ-AZ	0 (0)
Total		3,079 (100)

Abbreviations: AKI = Acute Kidney Injury, ICD = International Classification of Diseases

© 2019 James MT et al. *JAMA Network Open.*

eTable 3: Hazard ratios from multivariable Cox proportional hazards model for mortality and subhazard ratios from Fine and Grey competing risk models for nonfatal outcomes for demographic characteristics, comorbidities, and CKD, AKI, AKD without AKI and combinations of these states, among patients who received Scr testing in 2008

Variable	Hazard ratio (95% CI)	sub-Hazard ratio (95% CI)		
	Mortality	CKD development	CKD progression	ESKD
Age (per 1 year)	1.08 (1.08, 1.08)	1.05 (1.05, 1.06)	1.02 (1.02, 1.02)	0.99 (0.99, 0.99)
Male sex	1.34 (1.33, 1.36)	1.09 (1.08, 1.11)	1.15 (1.13, 1.17)	1.89 (1.76, 2.04)
Kidney disease categories				
NKD	Reference	Reference	N/A	Reference
CKD	1.37 (1.35, 1.39)	N/A	Reference	21.54 (19.44, 23.87)
AKD	1.42 (1.39, 1.45)	3.17 (3.1, 3.23)	N/A	8.56 (7.32, 10.01)
AKI	3.23 (3.16, 3.31)	1.32 (1.26, 1.38)	N/A	9.89 (8.12, 12.03)
CKD+AKD	1.92 (1.85, 2)	N/A	1.38 (1.33, 1.44)	65.95 (55.91, 77.8)
CKD+AKI	3.3 (3.19, 3.41)	N/A	0.98 (0.93, 1.04)	52.2 (43.2, 63.08)
Cancer	1.78 (1.75, 1.81)	0.94 (0.91, 0.98)	0.99 (0.95, 1.03)	1.14 (0.98, 1.34)
Myocardial infarction	1.18 (1.15, 1.2)	1.3 (1.25, 1.35)	1.22 (1.18, 1.27)	1.38 (1.19, 1.6)
Congestive heart failure	1.72 (1.69, 1.76)	1.52 (1.45, 1.58)	1.35 (1.29, 1.4)	1.9 (1.64, 2.2)
Cerebrovascular disease	1.36 (1.34, 1.39)	0.94 (0.91, 0.99)	1.07 (1.02, 1.12)	1.13 (0.94, 1.36)
Peripheral vascular disease	1.46 (1.41, 1.5)	1.22 (1.15, 1.29)	1.33 (1.25, 1.4)	2.11 (1.76, 2.53)
Chronic pulmonary disease	1.62 (1.6, 1.65)	0.94 (0.91, 0.96)	1.15 (1.12, 1.18)	0.95 (0.84, 1.08)
Dementia	2.53 (2.48, 2.58)	0.49 (0.46, 0.52)	0.59 (0.56, 0.62)	0.15 (0.09, 0.25)
Diabetes	1.01 (0.99, 1.04)	2.08 (2.03, 2.14)	1.22 (1.18, 1.27)	0.9 (0.76, 1.06)
Metastatic carcinoma	2.79 (2.63, 2.95)	0.73 (0.62, 0.85)	0.94 (0.78, 1.13)	0.51 (0.23, 1.15)
Liver disease (mild)	2.25 (2.14, 2.37)	1.2 (1.09, 1.31)	1.52 (1.35, 1.71)	2.2 (1.68, 2.9)
Liver disease (mod/severe)	4.28 (3.98, 4.61)	1.28 (1.08, 1.53)	2.25 (1.76, 2.87)	2.61 (1.72, 3.96)
Peptic ulcer disease	1.05 (0.99, 1.12)	1.02 (0.93, 1.11)	1.06 (0.95, 1.18)	1.22 (0.83, 1.79)
Rheumatic disease	1.28 (1.23, 1.33)	1.08 (1.01, 1.14)	1.45 (1.35, 1.56)	1.78 (1.39, 2.29)
Paraplegia and hemiplegia	1.68 (1.53, 1.84)	0.75 (0.61, 0.92)	1.26 (0.99, 1.6)	1.54 (0.84, 2.83)
Hypertension	0.89 (0.88, 0.91)	1.19 (1.17, 1.22)	1.01 (0.97, 1.04)	1.48 (1.33, 1.64)

Abbreviations: CKD = Chronic Kidney Disease, AKI = Acute Kidney Injury, AKD = Acute Kidney Diseases and Disorders, ESKD = End Stage Kidney Disease

© 2019 James MT et al. *JAMA Network Open.*

eTable 4: Unadjusted and adjusted outcomes restricted to Alberta residents with eGFR > 60 mL/min/1.73m2 prior to the index dates (excludes patients with no prior Scr measurements) who received Scr testing in 2008, according to CKD, AKI, AKD without AKI and combinations of these states

**Outcome**	**Events,** n (%)	**Total Follow-up,** Years	**Crude Hazard Ratio* (95% CI)**	**Age and Sex-adjusted Hazard Ratio† (95% CI)**	**Multivariable-adjusted Hazard Ratio* (95% CI)**
**Mortality (N= 797,594)**					
NKD	52,185 (7.4)	5,413,829	[Reference]	[Reference]	[Reference]
CKD+AKD w/o AKI	890 (34.0)	17,264	5.35 (5.00, 5.71)	1.83 (1.71, 1.95)	1.74 (1.62, 1.86)
CKD+AKI	596 (55.6)	5,156	11.88 (10.96, 12.88)	5.7 (5.26, 6.18)	3.99 (3.68, 4.33)
AKD without AKI	8,362 (24.7)	23,4371	3.7 (3.62, 3.79)	1.53 (1.5, 1.57)	1.39 (1.36, 1.43)
AKI	5,315 (48.3)	58,142	9.42 (9.16, 9.69)	4.82 (4.69, 4.96)	3.31 (3.22, 3.41)
CKD	5,808 (13.6)	318,121	1.89 (1.84, 1.95)	1.52 (1.48, 1.57)	1.48 (1.44, 1.52)
**CKD Development for those without CKD (N=751,196)**					
NKD	48,941 (6.9)	5,209,582	[Reference]	[Reference]	[Reference]
AKD without AKI	12,037 (35.6)	164,361	6.74 (6.6, 6.89)	3.91 (3.82, 4.00)	3.89 (3.8, 3.99)
AKI	1,524 (13.9)	50,012	2.16 (2.05, 2.28)	1.27 (1.21, 1.35)	1.27 (1.2, 1.35)
**CKD Progression for those with CKD (N=46,398)**					
CKD	11,292 (26.4)	280,869	[Reference]	[Reference]	[Reference]
CKD+AKD w/o AKI	1,122 (42.9)	13,563	1.86 (1.75, 1.98)	1.18 (1.1, 1.26)	1.15 (1.07, 1.23)
CKD+AKI	435 (40.6)	3,646	1.89 (1.7, 2.1)	1.29 (1.15, 1.45)	1.17 (1.05, 1.32)
**ESKD (N= 797,594)**					
NKD	345 (0.1)	5,413,032	[Reference]	[Reference]	[Reference]
CKD+AKD w/o AKI	74 (2.8)	17,088	58.06 (45.15, 74.65)	68.03 (51.57, 89.75)	58.27 (44, 77.16)
CKD+AKI	26 (2.4)	5,084	49.62 (33.24, 74.08)	54.2 (36.01, 81.58)	37.31 (24.31, 57.28)
AKD without AKI	114 (0.3)	23,4267	6.84 (5.54, 8.46)	8.15 (6.5, 10.22)	7.21 (5.72, 9.09)
AKI	64 (0.6)	58,170	11.78 (9.02, 15.39)	12.58 (9.53, 16.6)	8.58 (6.35, 11.59)
CKD	258 (0.6)	317,576	12.35 (10.51, 14.51)	12.06 (10.24, 14.2)	11.17 (9.46, 13.18)

*Full multivariable adjusted models adjusted for age, sex, socio-economic status, Aboriginal ethnicity, individual Charlson comorbidities, and hypertension.

† Hazard ratios were determined using Cox proportional hazards models for mortality, and subhazard ratios were determined using Fine and Gray competing risk models for CKD Development, CKD Progression, and ESKD

Abbreviations: CKD = Chronic Kidney Disease, AKI = Acute Kidney Injury, AKD = Acute Kidney Diseases and Disorders, ESKD = End Stage Kidney Disease

© 2019 James MT et al. *JAMA Network Open.*

eTable 5: Unadjusted and adjusted outcomes restricted to Alberta residents without albuminuria prior to the index dates (excludes patients with unmeasured, moderate or severe albuminuria) who received Scr testing in 2008, according to CKD, AKI, AKD without AKI and combinations of these states

Outcome	Events, n (%)	Total Follow-up, Years	Crude Hazard Ratio* (95% CI)	Age and Sex-adjusted Hazard Ratio† (95% CI)	Multivariable-adjusted Hazard Ratio* (95% CI)
**Mortality (N= 575,263)**					
NKD	29,079 (5.4)	412,2154	[Reference]	[Reference]	[Reference]
CKD+AKD w/o AKI	95 (66.0)	661	21.27 (17.39, 26.02)	3.31 (2.71, 4.05)	2.76 (2.26, 3.38)
CKD+AKI	380 (70.5)	2,146	26.2 (23.67, 28.99)	4.19 (3.79, 4.64)	3.41 (3.08, 3.78)
AKD without AKI	2,764 (21.5)	92,212	4.28 (4.12, 4.45)	1.28 (1.23, 1.34)	1.24 (1.19, 1.29)
AKI	1,662 (48.2)	18,328	13.04 (12.41, 13.7)	4.82 (4.58, 5.07)	3.13 (2.97, 3.29)
CKD	8,008 (32.2)	170,382	6.76 (6.6, 6.93)	1.26 (1.22, 1.29)	1.23 (1.19, 1.26)
**CKD Development for those without CKD (N= 549,730)**					
NKD	40,797 (7.6)	3,940,436	[Reference]	[Reference]	[Reference]
AKD without AKI	5,805 (45.1)	58,634	8.24 (8, 8.49)	3.34 (3.22, 3.45)	3.30 (3.19, 3.41)
AKI	787 (22.8)	14,225	3.52 (3.27, 3.8)	1.48 (1.36, 1.61)	1.37 (1.26, 1.5)
**CKD Progression for those with CKD (N= 25,533)**					
CKD	9,368 (37.7)	143,360	[Reference]	[Reference]	[Reference]
CKD+AKI	87 (60.4)	319	2.48 (1.9, 3.22)	2.25 (1.72, 2.95)	2.04 (1.55, 2.69)
CKD+AKD w/o AKI	227 (42.1)	1,486	1.27 (1.1, 1.46)	1.16 (1, 1.34)	1.07 (0.92, 1.25)
**ESKD (N= 575,263)**					
NKD	225 (0.04)	4,121,614	[Reference]	[Reference]	[Reference]
CKD+AKD w/o AKI	11 (0.7)	673	16.22 (2.28, 115.3)	17.41 (2.35, 128.69)	11.25 (1.47, 86.2)
CKD+AKI	15 (0.9)	2,168	21.82 (8.99, 52.94)	22.56 (8.81, 57.77)	17.20 (6.63, 44.6)
AKD without AKI	37 (0.3)	92,174	6.72 (4.74, 9.52)	6.94 (4.62, 10.43)	6.37 (4.2, 9.66)
AKI	20 (0.6)	18,369	13.37 (8.45, 21.15)	13.54 (8.31, 22.05)	9.08 (5.26, 15.68)
CKD	125 (0.5)	170,145	11.70 (9.4, 14.56)	12.38 (8.62, 17.8)	11.42 (7.82, 16.68)

*Full multivariable adjusted models adjusted for age, sex, socio-economic status, Aboriginal ethnicity, individual Charlson comorbidities, and hypertension.

† Hazard ratios were determined using Cox proportional hazards models for mortality, and subhazard ratios were determined using Fine and Gray competing risk models for CKD Development, CKD Progression, and ESKD

Abbreviations: CKD = Chronic Kidney Disease, AKI = Acute Kidney Injury, AKD = Acute Kidney Diseases and Disorders, ESKD = End Stage Kidney Disease

© 2019 James MT et al. *JAMA Network Open.*

eTable 6: Unadjusted and adjusted outcomes for Alberta residents who received Scr testing in 2008, using an alternate definition for AKD requiring an index eGFR < 60 ml/min/1.73m^2 with a decline > 10 mL/min/1.73m^2 from previous baseline or subsequent increase in Scr by 50% within 7 days to 3 months

Outcome	Events, n (%)	Total Follow-up, Years	Crude Hazard Ratio* (95% CI)	Age and Sex-adjusted Hazard Ratio* (95% CI)	Multivariable-adjusted Hazard Ratio* (95% CI)
**Mortality (N= 1,109,099)**					
NKD	69,824 (7.5)	7,093,412	[Reference]	[Reference]	[Reference]
CKD+AKD w/o AKI	2,532 (49.0)	29,722	8.63 (8.29, 8.97)	2.16 (2.08, 2.25)	1.99 (1.91, 2.07)
CKD+AKI	3,585 (71.5)	19,574	18.33 (17.73, 18.96)	4.19 (4.05, 4.34)	3.30 (3.19, 3.41)
AKD without AKI	8,771 (26.3)	226,859	3.92 (3.84, 4.01)	1.68 (1.64, 1.72)	1.57 (1.54, 1.61)
AKI	7,964 (50.5)	80,864	9.92 (9.69, 10.15)	4.47 (4.36, 4.57)	3.23 (3.16, 3.31)
CKD	35,261 (29.5)	811,360	4.41 (4.36, 4.47)	1.42 (1.4, 1.44)	1.37 (1.35, 1.39)
**CKD Development for those without CKD (N=979,380)**					
NKD	72,515 (7.8)	6,765,142	[Reference]	[Reference]	[Reference]
AKD without AKI	11,724 (35.1)	159,241	5.77 (5.65, 5.89)	3.04 (2.97, 3.11)	2.98 (2.91, 3.05)
AKI	3,066 (19.4)	65,813	2.83 (2.73, 2.94)	1.34 (1.28, 1.4)	1.24 (1.18, 1.29)
**CKD Progression for those with CKD (N=129,719)**					
CKD	41,453 (34.7)	686,404	[Reference]	[Reference]	[Reference]
CKD+AKD w/o AKI	2,577 (49.9)	21,687	1.7 (1.63, 1.77)	1.43 (1.37, 1.49)	1.39 (1.33, 1.45)
CKD+AKI	2,064 (41.2)	13,488	1.37 (1.31, 1.44)	1.06 (1.01, 1.12)	0.98 (0.93, 1.03)
**ESKD (N= 1,109,099)**					
NKD	679 (0.1)	7,091,769	[Reference]	[Reference]	[Reference]
CKD+AKD w/o AKI	218 (4.2)	29,174	58.45 (50.17, 68.09)	74.74 (63.08, 88.55)	65.64 (55.26, 77.98)
CKD+AKI	176 (3.5)	19,195	48.68 (41.21, 57.49)	64.70 (53.84, 77.75)	49.65 (41.13, 59.94)
AKD without AKI	210 (0.6)	226,469	8.58 (7.35, 10.01)	9.97 (8.5, 11.68)	9.00 (7.66, 10.57)
AKI	135 (0.9)	80,752	11.65 (9.69, 14.02)	12.71 (10.52, 15.36)	9.50 (7.81, 11.55)
CKD	1,628 (1.4)	806,911	18.6 (17.01, 20.34)	22.01 (19.92, 24.3)	20.94 (18.92, 23.16)

* Hazard ratios were determined using Cox proportional hazards models for mortality, and subhazard ratios were determined using Fine and Gray competing risk models for CKD Development, CKD Progression, and ESKD

Abbreviations: CKD = Chronic Kidney Disease, AKI = Acute Kidney Injury, AKD = Acute Kidney Diseases and Disorders, ESKD = End Stage Kidney Disease

© 2019 James MT et al. *JAMA Network Open.*

eTable 7: Categorical net reclassification improvement (NRI) and integrated discrimination improvement (IDI) with addition of AKD criteria to kidney disease classification for Alberta residents who received Scr testing in 2008, according to CKD, AKI, AKD and combinations of these states

Outcome	Comparisons*	NRI events, n (%)	Non-NRI events, n (%)	Overall NRI, % [95%CI]	IDI, % (95%CI)
**Mortality**	AKI, CKD, AKD, CKD+AKI, CKD+AKD versus AKI, CKD, CKD+AKI	139 (0.11)	3140 (0.32)	0.43 (0.31, 0.54)	0.29 (0.26, 0.32)
**CKD development for those without CKD**	AKI, AKD versus AKI	-1,358 (-1.55)	29,831 (3.34)	1.79 (0.46, 3.11)	3.07 (0.35, 5.79)
**CKD progression for those with CKD**	CKD+AKI, CKD+AKD versus CKD+AKI	36 (0.08)	359 (0.42)	0.51 (0.38, 0.65)	0.33 (0.27, 0.39)
**ESKD**	AKI, CKD, AKD, CKD+AKI, CKD+AKD versus AKI, CKD, CKD+AKI	378 (12.40)	-20,434 (-1.85)	10.56 (7.84, 13.15)	0.61 (0.39, 0.84)

*All models included age, sex, socioeconomic status, Aboriginal ethnicity, hypertension, and each Charlson comorbidity as independent variables in addition to the kidney disease classifications included in the comparisons

The NRI and IDI were estimated at 8 years follow-up time. Categorical NRI risk categories were <1%, 1-5%, 5-10%, 10-20% and >20%

© 2019 James MT et al. *JAMA Network Open.*

eFigure 1: Time frames and sequence of steps for using serum creatinine / estimated glomerular filtration rate and albuminuria determinations to characterize NKD, CKD, AKD, AKI.

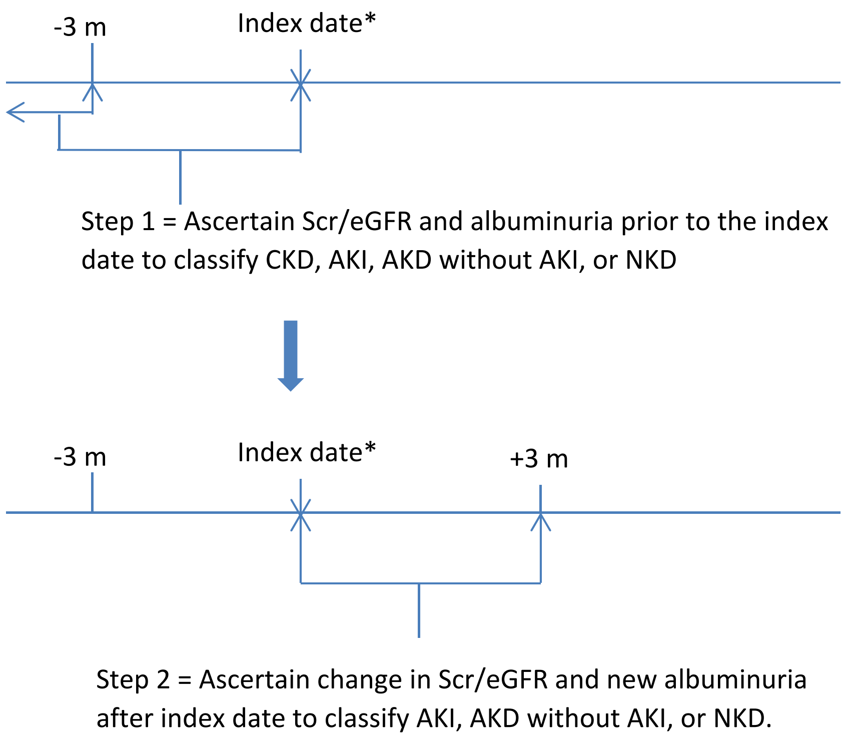

*The index date was defined by each patient's first serum creatinine / estimated glomerular filtration rate determination in 2008.
Abbreviations: NKD = No Kidney Disease, CKD = Chronic Kidney Disease, AKI = Acute Kidney Injury, AKD = Acute Kidney Diseases and Disorders.

© 2019 James MT et al. *JAMA Network Open.*

eFigure 2: Study cohort formation

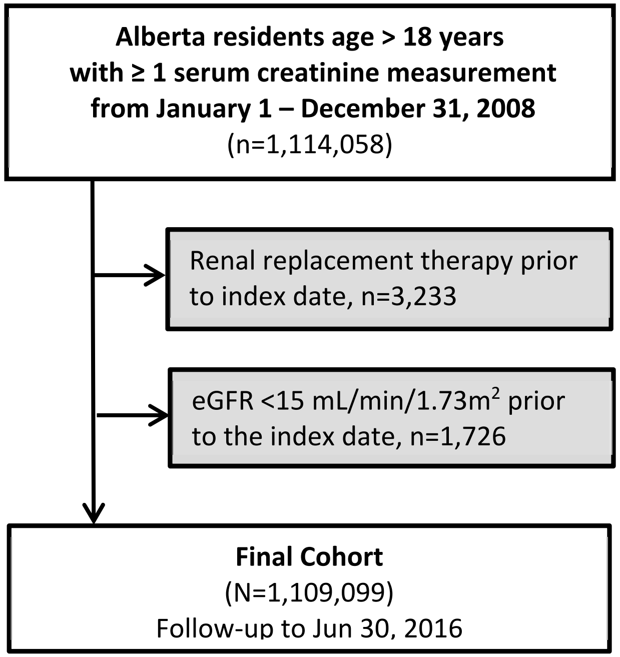

© 2019 James MT et al. *JAMA Network Open.*

eFigure 3: Numbers of participants with AKD based on meeting one of more of each of the criterion used for AKD identification

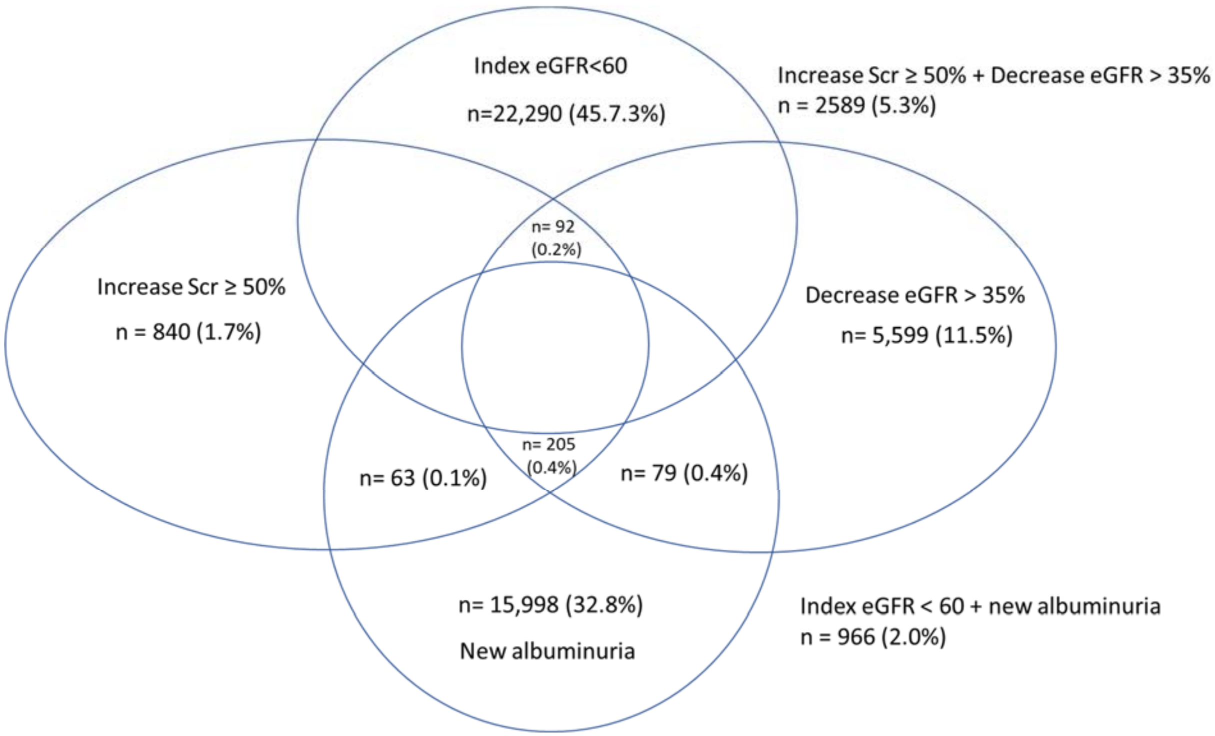

*The number and percentage or study participants based on combinations of the AKD criterion are shown for all scenarios that comprised >0.1% of all patients with AKD.

© 2019 James MT et al. *JAMA Network Open.*
